# Alkaline pH Increases Swimming Speed and Facilitates Mucus Penetration for Vibrio cholerae

**DOI:** 10.1128/JB.00607-20

**Published:** 2021-03-08

**Authors:** Nguyen T. Q. Nhu, John S. Lee, Helen J. Wang, Yann S. Dufour

**Affiliations:** a Department of Microbiology and Molecular Genetics, Michigan State University, East Lansing, Michigan, USA; Brigham and Women's Hospital/Harvard Medical School

**Keywords:** *Vibrio cholerae*, intestinal mucus, flagellar motility, pH, microrheology, cell tracking

## Abstract

Intestinal mucus is the first line of defense against intestinal pathogens. It acts as a physical barrier between epithelial tissues and the lumen that enteropathogens must overcome to establish a successful infection. We investigated the motile behavior of two Vibrio cholerae strains (El Tor C6706 and Classical O395) in mucus using single-cell tracking in unprocessed porcine intestinal mucus. We determined that V. cholerae can penetrate mucus using flagellar motility and that alkaline pH increases swimming speed and, consequently, improves mucus penetration. Microrheological measurements indicate that changes in pH between 6 and 8 (the physiological range for the human small intestine) had little effect on the viscoelastic properties of mucus. Finally, we determined that acidic pH promotes surface attachment by activating the mannose-sensitive hemagglutinin (MshA) pilus in V. cholerae El Tor C6706 without a measurable change in the total cellular concentration of the secondary messenger cyclic dimeric GMP (c-di-GMP). Overall, our results support the hypothesis that pH is an important factor affecting the motile behavior of V. cholerae and its ability to penetrate mucus. Therefore, changes in pH along the human small intestine may play a role in determining the preferred site for V. cholerae during infection.

**IMPORTANCE** The diarrheal disease cholera is still a burden for populations in developing countries with poor sanitation. To develop effective vaccines and prevention strategies against Vibrio cholerae, we must understand the initial steps of infection leading to the colonization of the small intestine. To infect the host and deliver the cholera toxin, V. cholerae has to penetrate the mucus layer protecting the intestinal tissues. However, the interaction of V. cholerae with intestinal mucus has not been extensively investigated. In this report, we demonstrated using single-cell tracking that V. cholerae can penetrate intestinal mucus using flagellar motility. In addition, we observed that alkaline pH improves the ability of V. cholerae to penetrate mucus. This finding has important implications for understanding the dynamics of infection, because pH varies significantly along the small intestine, between individuals, and between species. Blocking mucus penetration by interfering with flagellar motility in V. cholerae, reinforcing the mucosa, controlling intestinal pH, or manipulating the intestinal microbiome will offer new strategies to fight cholera.

## Abstract (precis)

The diarrheal disease cholera is still a burden for populations in developing countries with poor sanitation. To develop effective vaccines and prevention strategies against Vibrio cholerae, we must understand the initial steps of infection leading to the colonization of the small intestine.

## INTRODUCTION

Vibrio cholerae is the cause of an ongoing cholera pandemic, with up to 4 million cases per year from regions of the world that do not have access to potable water (1). Without proper rehydration and antibiotic treatments, severe diarrhea triggered by the cholera toxin can be fatal (2). Preventive measures and vaccines against V. cholerae have had partial success (3, 4), but cholera outbreaks are still a significant burden for populations living in developing regions or after natural disaster, such as Bangladesh and Haiti (1).

V. cholerae is represented by more than 200 serogroups that are endemic to sea and brackish waters and often found associated with copepods (5, 6). However, only the O1 and O139 serogroups have been associated with cholera, the diarrheal disease in humans (7). Within the O1 serogroup, the Classical biotype dominated the first 6 recorded cholera pandemics. The ongoing 7th pandemic is dominated by the El Tor biotype, which has rapidly displaced the Classical biotype in the environment (8, 9). Although similar, the two biotypes have differences in their genetic makeups, signaling dynamics, and behaviors (10–12). The relative importance of these unique traits has not been fully elucidated yet.

V. cholerae colonizes the mucus of the small intestine without invading epithelial cells. When reaching the intestinal crypts, V. cholerae secretes the cholera toxin, which targets epithelial cells to activate the chlorine channel proteins and consequently trigger a massive efflux of chlorine ions and water into the intestinal lumen. Many aspects of V. cholerae physiology and the regulation of virulence factor expression have been investigated to recapitulate the dynamics of infection after ingestion (13–15), such as pilus production (16), type 6 secretion system (17), quorum sensing (18), biofilm formation (19), and flagellar motility (20). While these different behaviors have been shown to contribute to V. cholerae success during infection, the specific sequence of events and site-specific activities in the intestine are still under investigation.

Flagellar motility is essential for V. cholerae infection. Studies of transcription profiles and screens of mutant libraries during the infection of animal models and humans identified genes involved in chemotaxis and motility functions (21). Nonmotile V. cholerae mutants have reduced virulence and intestinal colonization (22–24). In addition, previous work supports the hypothesis that protective immunity is mostly provided by mucosal antibodies that inhibit V. cholerae motility through bivalent binding of the O-antigen (25). Motility may not be required for survival and growth in the intestine, since nonmotile mutants do not appear to suffer a large competitive disadvantage when inoculated with motile V. cholerae (26). However, flagellar motility is likely necessary to penetrate the mucus layer protecting the intestinal tissue and reach epithelial cells to deliver the cholera toxin.

Mucus is a complex hydrogel made of mucins (2 to 10%, wt/vol), lipids, and DNA (27) and is difficult for motile bacteria to penetrate. Mucins are large and highly glycosylated proteins cross-linked by disulfide bonds reinforced by hydrophobic interactions to form a tight mesh. The intestinal mucus layer is continuously renewed by secretion of highly O-glycosylated MUC2 mucin by goblet cells (240 ± 60 μm per hour) (28). Consequently, mucus forms a selective diffusion barrier undergoing continuous regeneration, the rate of which can increase in response to threats such as the cholera toxin (29). Histological analyses revealed that the inner part of the mucus layer is mostly free of bacteria (30). In the small intestine, the mucus layer is thinner in the proximal part (∼200 μm) than the distal part (∼500 μm) (31). These observations raise questions of how V. cholerae can penetrate mucus and why it preferably infects the distal small intestine where the mucosa is thicker.

Few studies have directly observed the motile behavior of individual bacteria in mucus to characterize the strategy used to compromise the protective layer. Helicobacter pylori, which colonizes the thick mucus layer of the stomach, facilitates flagellar motility through mucus by enzymatically increasing the local pH to liquefy the mucus gel structure (32, 33). It is also believed that the helical cell shape of both H. pylori and Campylobacter jejuni, which colonizes the thick mucus layer of the cecum, facilitates mucus penetration by allowing the body to push against the mucin matrix like a corkscrew (34, 35). Recent work demonstrated that the peritrichous rod-shaped bacteria Escherichia coli and Bacillus subtilis can penetrate cervical mucus by taking advantage of water channels created by shear forces during secretion (36). The behavior of V. cholerae in mucus has not been described.

In this study, we characterized the behavior of individual cells from two V. cholerae strains in unprocessed porcine intestinal mucus and tested if V. cholerae alters the rheological properties of mucus over time. We demonstrated that V. cholerae can swim through porcine intestinal mucus even without measurable changes in mucus rheology and measured that porcine intestinal mucus is not sensitive to change in pH between 6 and 8. However, alkaline conditions dramatically increase swimming speed and mucus penetration for V. cholerae. These results shed light on how V. cholerae can overcome the defensive mucus layer and the role of intestinal pH during the initial stage of infection.

## RESULTS

### V. cholerae can penetrate intestinal mucus using flagellar motility.

**FIG 1:**
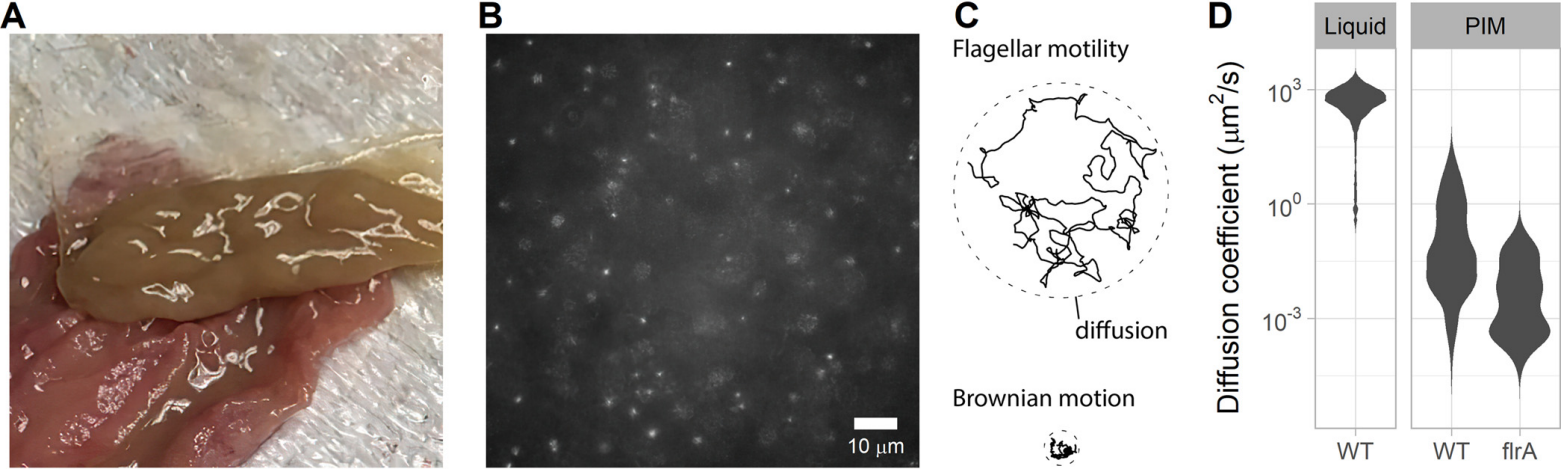
V. cholerae Classical O395 flagellar motility through unprocessed porcine intestinal mucus (PIM). (A) Mucus scraped from the medial part of the small intestine of an adult pig. (B) Representative epifluorescence image at ×40 magnification of V. cholerae Classical O395 (expressing the green fluorescent protein) swimming in unprocessed pig intestinal mucus between two glass coverslips. (C) Motile cells can be distinguished from nonmotile by comparing the trajectories of effective diffusion coefficients. (D) Distributions of diffusion coefficients from individual trajectories in liquid and PIM. Motile wild-type V. cholerae O395 (WT) was compared to a nonmotile mutant (*flrA*) in PIM. Each distribution represents 3 to 12 replicates combining between 500 and 6,000 individual trajectories (between 250 and 1,700 min of cumulative time).

We tracked fluorescently labeled V. cholerae Classical O395 in unprocessed mucus that was scraped from the medial part of the small intestine of an adult pig (Fig. 1A and B). Porcine mucus has been shown to be the most comparable to human mucus regarding structure and thickness compared to several animal models and also acts as a physical barrier between intestinal tissues and bacteria in the lumen (37, 38). As expected, the movement of V. cholerae, as quantified by the trajectory effective diffusion coefficient (Fig. 1C), is severely impaired in mucus compared to swimming in a liquid environment (Fig. 1D). To determine the proportion of cells using flagellar motility to swim through mucus, we also measured the effective diffusion coefficient of nonflagellated cells (*flrA* mutant) and determined that a diffusion coefficient above 10^−0.5^ μm^2^/s was evidence of flagellar motility (Fig. 1D). Most wild-type cells (∼75%) were trapped and unable to swim through the mucus mesh. The rest of the population (∼25%) was able to swim through the mucus while being caught in the mucus mesh only intermittently. Because cells were not moving freely and did not have a constant diffusion coefficient, the reported diffusion coefficient represents an average over the entire length of each trajectory.

### Alkaline pH improves the motility of V. cholerae in intestinal mucus.

**FIG 2:**
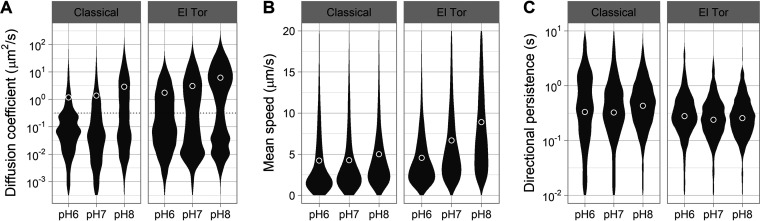
Effects of pH on the motility of V. cholerae through porcine intestinal mucus. (A) Distributions of diffusion coefficient from individual trajectories in mucus buffered at different pH. Cells with a diffusion coefficient of <10^−0.5^ μm^2^/s were categorized as nonmotile or trapped and were excluded from the following analyses. (B) Distributions of swimming speed from the motile cell populations. (C) Distributions of directional persistence time scales from the motile cell populations. Each distribution represents 8 to 12 replicates combining between 6,000 and 19,000 individual trajectories (between 1,000 and 2,600 min of cumulative time). Circles indicate means for the motile populations.

V. cholerae appears to colonize preferentially the lower part of the small intestine (ileum), where the mucus layer is thicker (31). The ileum is also the most alkaline region of the small intestine (pH 7 to 8), whereas the jejunum (upper part) is slightly acidic (pH 6 to 7) (39). Therefore, we tested if pH influenced the motile behavior of V. cholerae in intestinal mucus. We equilibrated unprocessed porcine intestinal mucus with phosphate saline buffer at pH 6, 7, and 8. We then tracked the swimming behavior of both V. cholerae Classical O395 and El Tor C6706 in mucus at each pH. The proportions of swimming cells and the swimming speeds increased as pH increased for both strains (*P*  < 10^−4^) (Fig. 2A and B). At pH 8, 51% of Classical 0395 and 76% of El Tor C6706 organisms could swim through the mucus. Directional persistence (the time scale at which cells change direction) did not show a response, indicating that the reversal frequency of the flagellar motor was not affected by the change in pH (Fig. 2C). Overall, alkaline pH improves the motility of V. cholerae in mucus, but pH could be affecting either the rheological properties of mucus or the physiology of V. cholerae.

### Change in pH between 6 and 8 had little effect on the mucus rheological properties.

**FIG 3:**
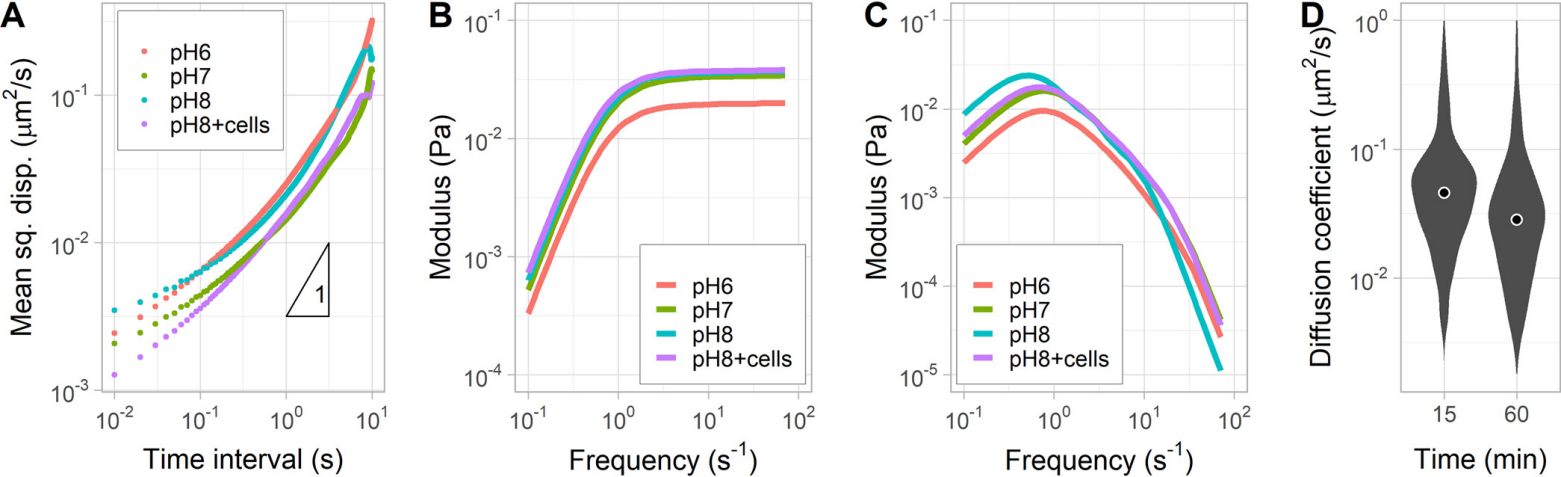
Passive microrheology of porcine intestinal mucus. (A) Mean-squared displacement (mean sq. disp.) of PEG-coated 1-μm polystyrene beads with respect to time at different pHs (represented by different colors) and after incubation with V. cholerae. The data points (circles) are the average trajectories from 4 to 6 replicates (10 to 25 individual trajectories). A polynomial fit to the data was used to calculate the storage and loss moduli using the generalized Stokes-Einstein relation. (B) Storage moduli (elasticity) of porcine intestinal mucus at different pHs. (C) Loss moduli (viscosity) of porcine intestinal mucus at different pHs. (D) Distributions of the diffusion coefficient of nonmotile V. cholerae (*flrA*) after incubation in mucus at pH 8. Each distribution represents 6 replicates combining between 1,000 and 2,000 individual trajectories (∼150 min of cumulative time). Circles indicate means.

To test if pH affects the structure of mucus, we tracked the motion of 1-μm fluorescent polystyrene beads coated with polyethylene glycol that were mixed in the same mucus samples used to track V. cholerae. The thermally driven diffusive behavior of beads is affected by the viscoelastic properties of mucus. The 1-μm beads had a subdiffusive behavior (slope of the mean squared displacement [MSD] of <1), indicating that the motion of the beads was constrained by the mucin matrix (Fig. 3A) (27). The mucin matrix pore sizes were previously estimated to be ∼240 nm using electron microscopy (28, 40). Consequently, the diffusive motion of 1-μm beads and similarly sized bacteria, such as V. cholerae, is severely diminished in mucus.

The loss (viscous) and storage (elastic) moduli of the mucus can be calculated from mean squared displacement of the beads with respect to time using the generalized Stokes-Einstein relation (41). This analysis indicated that the viscosity and elasticity of the porcine intestinal mucus did not change substantially when pH was equilibrated at 6, 7, or 8 (Fig. 3B and C). We also determined that a prolonged incubation (1 h) of mucus with V. cholerae El Tor C6706 had no measurable effect on the mucus rheology at pH 8. The average diffusion coefficient of nonmotile V. cholerae (*flrA*) decreased slightly after 1 h in mucus compared to that at 15 min (Fig. 3D). Incubation of mucus with V. cholerae El Tor C6706 at pH 6 and 7 produced identical results (see Fig. S1 in the supplemental material). Therefore, we concluded that the improved motility of V. cholerae in mucus at pH 8 is likely not attributed to changes in the mucus structure.

Previous studies have characterized the behavior of V. cholerae in mucus reconstituted from commercially available purified mucin (42, 43). We characterized the rheological properties of solutions of mucins from bovine submaxillary glands and porcine stomach purchased commercially. We used a 3% (wt/vol) concentration, which is comparable to native mucus (27, 44), in phosphate saline buffer at pH 8. The beads had purely diffusive trajectories, indicating that the solutions were viscous but not elastic (Fig. S2A). The storage and loss moduli of the purified mucin solutions were lower than those of our porcine mucus sample (Fig. S2B and C). Therefore, the purified mucins failed to reconstitute the gel structure of native mucus when dissolved in solution, likely because they do not spontaneously cross-link. This result indicates that the physical structure of mucus reconstituted from purified mucins is not comparable to unprocessed mucus.

### Alkaline pH promotes the spread of V. cholerae colonies in soft agar.

**FIG 4:**
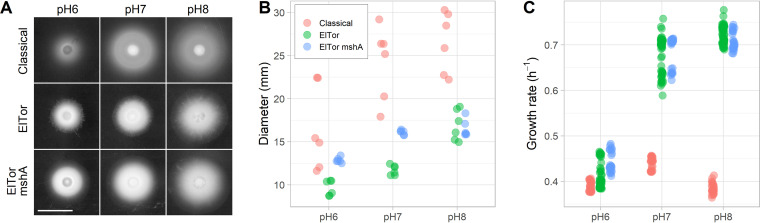
Effects of pH on the spreading of V. cholerae colonies in soft agar. (A) Representative colonies from the Classical O395 and El Tor C6706 (wild type and *mshA*) at different pHs (bar is 10 mm). (B) Measured colony diameters at different pHs for all experimental replicates. (C) Measured growth rates in batch cultures at different pHs for all experimental replicates.

To test the effect of pH on V. cholerae motility in the traditional soft-agar assay, we measured the spread of colonies in M9 salts supplemented with pyruvate, tryptone, and 0.3% (wt/vol) agar (Fig. 4A). Both Classical 0395 and El Tor C6706 formed significantly larger colonies at alkaline pH (*P*  < 10^−4^) (Fig. 4B). The colony morphology of El Tor C6706 was denser and rugged at the edge compared to that of Classical 0395. One of the differences between the two strains is that Classical does not elaborate the MshA (mannose-sensitive hemagglutinin) pilus that mediates cell attachment (45–47). We inactivated *mshA* in the El Tor background to test if MshA affects colony morphology (Fig. 4A). The colonies of the *mshA* mutant had smoother edges and spread further (*P*  < 10^−4^) (Fig. 4B) but remained dense like the wild type. Overall, V. cholerae spreads further in soft agar at alkaline pH.

Colony spreading is a function of cell motility and chemotaxis to self-generated chemical gradients but also a function of growth rate (48–50). V. cholerae growth is known to be sensitive to acidic pH (51). Therefore, we also measured growth rates in batch cultures at pH 6, 7, and 8 in M9 salts supplemented with pyruvate at 37°C (Fig. 4C). At neutral pH, El Tor C6706 grew ∼60% faster (63-min generation time) than Classical O395 (98-min generation time). pH had only a small effect on the generation time of Classical O395. El Tor C6706 grew fastest at pH 7 and 8 (63-min and 59-min generation times) but significantly slower at pH 6 (103 min) (*P*  < 10^−4^). The expression of MshA had a very small but measurable effect on the generation time of El Tor C6706. The effect of pH on growth rate may explain why colony spreading was reduced for El Tor C6706. However, these results do not explain why Classical O395 was similarly affected by pH and spread faster than El Tor C6706 in soft agar. Therefore, we hypothesized that pH affects V. cholerae flagellar motility directly.

### V. cholerae swims faster at alkaline pH.

To characterize how the swimming behavior of V. cholerae is affected by pH more directly, we tracked single cells swimming in a liquid environment between 2 glass coverslips (∼10 μm in height). The diffusion coefficient of 1-μm beads and nonmotile cells (*flrA* mutant) is distributed between 0.1 and 10 μm^2^/s in liquid. Therefore, trajectories with an effective diffusion coefficient below 10 μm^2^/s were categorized as nonmotile in the different conditions tested and excluded from the calculations of swimming parameters.

**FIG 5:**
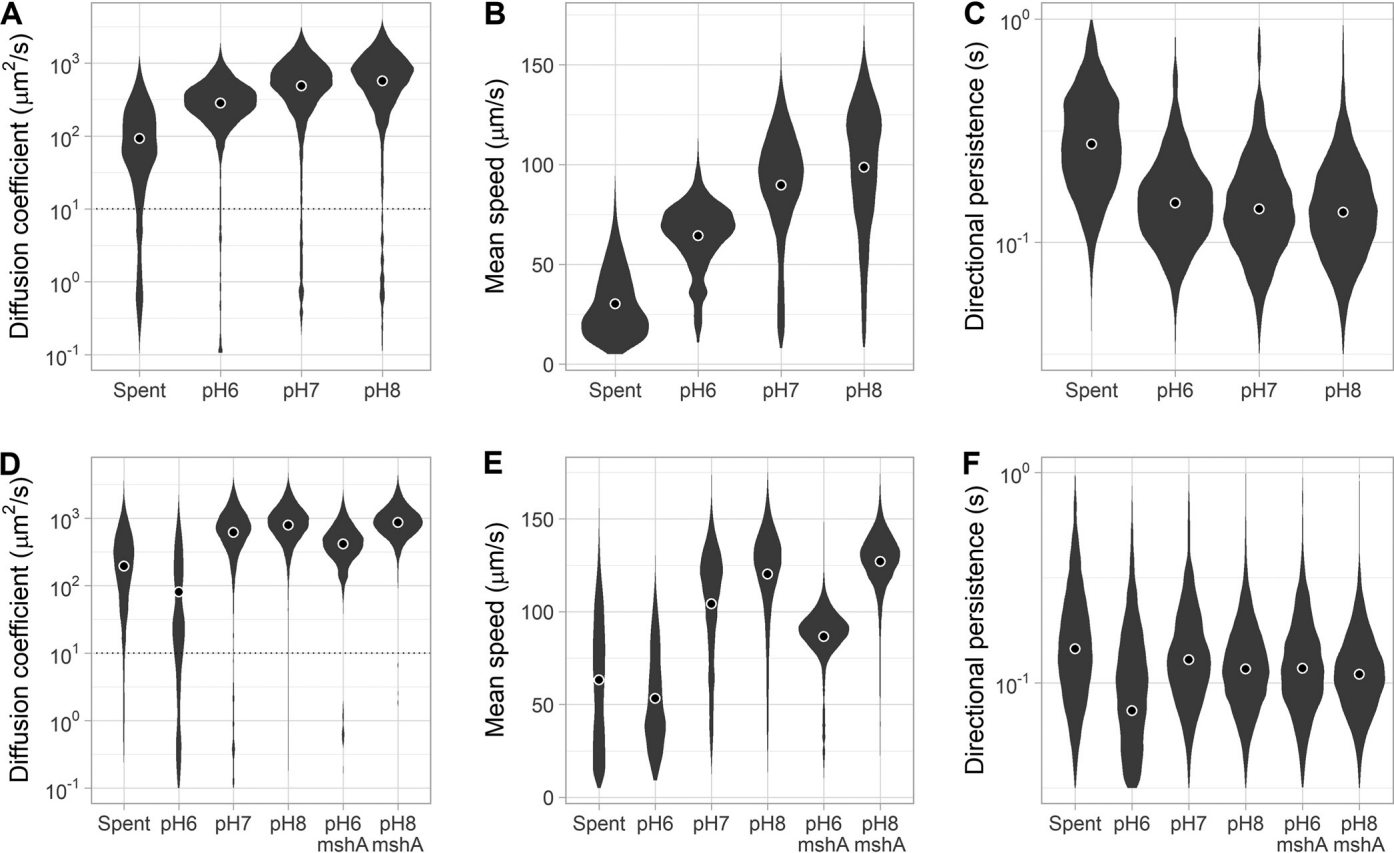
Effects of pH on V. cholerae flagellar motility. (A) Distributions of diffusion coefficient of Classical O395 from single-cell trajectories in spent medium (Spent) or in fresh medium at different pHs. Trajectories below 10 μm^2^/s were categorized as nonmotile and excluded from the remaining analyses. (B) Distributions of swimming speed from the motile cell populations. (C) Distributions of trajectory directional persistence from the motile cell populations. (D) Distributions of diffusion coefficients of El Tor C6706 from single-cell trajectories in spent medium (Spent) or in fresh medium at different pHs. An *mshA* mutant was also tracked. (E) Distributions of swimming speed from the motile cell populations. (F) Distributions of trajectory directional persistence from the motile cell populations. Each distribution represents 3 replicates combining between 2,000 and 10,000 individual trajectories (between 100 and 500 min of cumulative time). Circles indicate means for the motile populations.

For Classical O395, most cells were highly motile near the end of the exponential growth phase. The diffusion coefficient and swimming speed of the motile population increased upon transfer from the spent growth medium to fresh medium at all pHs (*P*  < 10^−4^), likely because of the replenishment of the energy source (addition of pyruvate to spent medium had an identical effect; data not shown). In fresh medium, Classical O395 was most diffusive at alkaline pH (*P*  < 10^−4^) (Fig. 5A). Both swimming speed and the frequency at which cells change direction by reversing the flagellar motor rotation affects diffusion coefficient. However, analysis of the trajectories revealed that only swimming speed was affected by pH (*P*  < 10^−4^) (Fig. 5B). On the other hand, the directional persistence of the cell trajectories did not change substantially, indicating that the reversal frequency of the flagellar motor was not affected by pH in Classical O395 (Fig. 5C).

Tracking of El Tor C676 revealed a more complex behavioral response to change in pH. Upon transfer from the growth medium to pH 6, two-thirds of the population became nonmotile (Fig. 5D), while at pH 7 and 8 the response was like that of Classical O395. We hypothesized that MshA-mediated surface attachment was activated in El Tor C6706 at acidic pH, so we also tracked the swimming behavior of an *mshA* mutant at pH 6 and 8. The *mshA* mutant was fully motile at pH 6 (Fig. 5D); thus, we concluded that El Tor C6706 activates MshA-mediated attachment at acidic pH but not at neutral or alkaline pH under our growth conditions. These results are consistent with the observation that the presence of MshA reduces the spread of colonies on soft agar (Fig. 4). In the absence of MshA, El Tor C6706 swimming speed more than doubled between pH 6 and 8 (*P*  < 10^−4^) (Fig. 5E), while the directional persistence was unaffected (Fig. 5F).

The second messenger c-di-GMP regulates many behavioral responses in V. cholerae, including flagellar motility and surface attachment (52–54). To test if the cytoplasmic c-di-GMP concentration changes after a shift in pH, we quantified the bulk c-di-GMP concentrations after transfer to buffer solution at different pH using mass spectrometry with El Tor C6706 sampled during the early stationary phase. No measurable change in the total c-di-GMP concentration could be attributed to a change in pH (Fig. S3). Our results cannot exclude the possibility that pH activates c-di-GMP signaling through localized pathways, as previously demonstrated in V. cholerae (55) and Escherichia coli (56), or that c-di-GMP changed and returned to the prestimulus concentrations during the incubation period (15 min). Overall, the increase in swimming speed in both V. cholerae strains is likely the main factor underlying improved motility in intestinal mucus and soft agar at alkaline pH.

### Inhibiting Na^+^-NQR in V. cholerae reduces swimming speed and hydrogel penetration.

V. cholerae uses a sodium motive force to power its flagellar motor (57). Therefore, change in pH is unlikely to have a direct effect on the flagellar motor torque and rotation speed in V. cholerae. However, maintaining a strong sodium gradient across the cell membrane when the motor is rotating at high speed is energetically costly (58). V. cholerae uses several sodium transporters, but most of the sodium export is done by the NADH:quinone oxidoreductase (Na^+^-NQR) as part of the respiratory chain (59). The activity of the Na^+^-NQR pump is strongest at alkaline pH, while cells are respiring (60). Previous studies showed that Vibrio alginolyticus is unable to maintain a strong sodium potential across the cell membrane when the cell environment becomes acidic (61). Therefore, the reduction of swimming speed we observed at acidic pH is likely the result of the reduction of the Na^+^-NQR pump activity.

To test if Na^+^-NQR activity plays a role in the ability of V. cholerae to penetrate mucus, we added 2-*n*-heptyl-4-hydroxyquinoline *N*-oxide (HQNO), a strong inhibitor of Na^+^-NQR activity (57). Unfortunately, mucus has a strong binding affinity to HQNO, which becomes unavailable to inhibit the Na^+^-NQR pump. Mucus has been previously shown to bind similar small molecules with high affinity (62). Buffer containing 100 μM HQNO recovered after incubation with porcine intestinal mucus had no effect on V. cholerae swimming speed or behavior.

**FIG 6:**
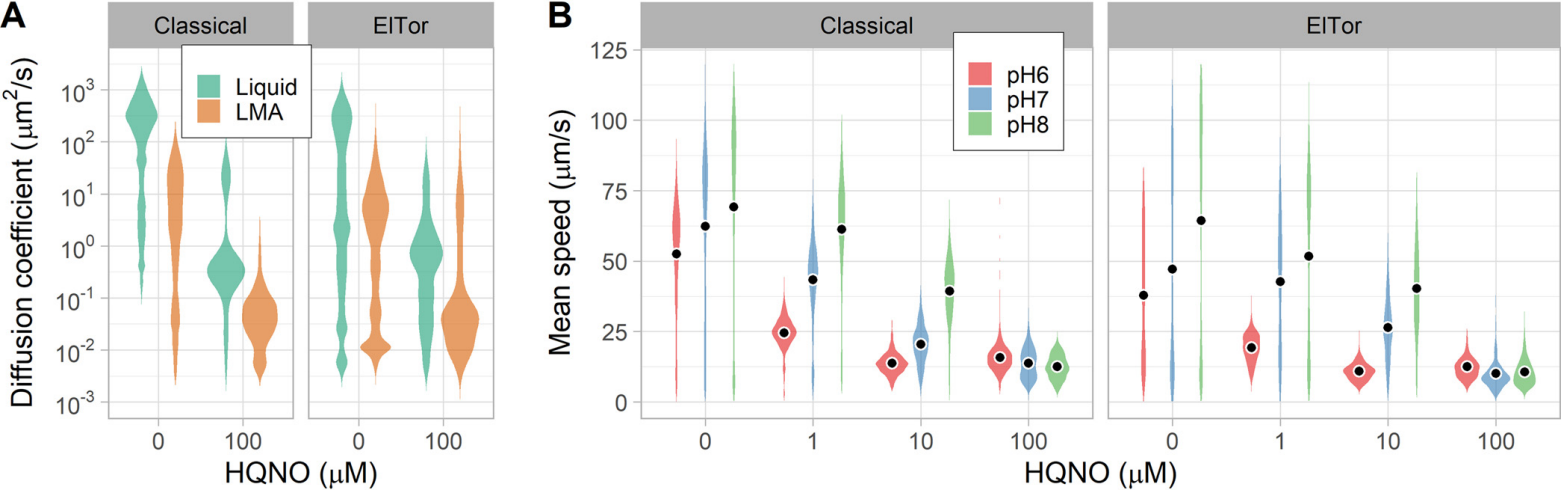
Effects of inhibiting the Na^+^-NQR pump on flagellar motility in V. cholerae. (A) Distributions of diffusion coefficient in liquid and 0.3% (wt/vol) agarose buffered at pH 8 and with the addition of 100 μM HQNO. Each distribution represents 6 replicates combining between 1,000 and 3,000 individual trajectories (∼1,000 min of cumulative time). (B) Distributions of swimming speed at different pHs as a function of HQNO concentration. Each distribution represents at least 6 replicates combining between 2,000 and 6,000 individual trajectories (between 500 and 1,000 min of cumulative time). Circles indicate means for the motile populations.

Instead, we tested the effect of HQNO on V. cholerae motility in liquid and agarose gel at pH 8. Low-melting-temperature agarose at 0.3% (wt/vol) forms a hydrogel similar to our porcine intestinal mucus samples but with larger mesh pores and less viscosity and elasticity (Fig. S2). As observed with mucus, agarose gel impaired the motility of V. cholerae but did not completely abolish it (as expected from the soft-agar plate assays). HQNO did not appear to interact with agarose, as it dramatically reduced the effective diffusion coefficients of both V. cholerae strains (*P*  < 10^−4^) (Fig. 6A). Most cells were unable to swim through the agarose gel in the presence of HQNO (diffusion coefficient of <10^−0.5^ μm^2^/s), supporting the hypothesis that the ability to maintain a strong sodium gradient is required for V. cholerae to escape the gel matrix using flagellar motility.

To test that HQNO did not block rotation of the flagellar motor, we characterized the dose response of V. cholerae swimming speed at low viscosity (in liquid). The swimming speed of the motile cell population decreased in a dose-dependent manner with increasing concentrations of HQNO (Fig. 6B). The effect was more pronounced at acidic pH, indicating a synergistic interaction between the effect of low pH and HQNO binding in the pump channel. Swimming speed was very low at 100 μM HQNO, but both strains were still motile. Overall, our results are consistent with a model that links the reduced activity of the Na^+^-NQR pump at acidic pH to the observed reduction in swimming speed and motility in porcine intestinal mucus.

## DISCUSSION

In this work, we demonstrated that V. cholerae can penetrate intestinal mucus using flagellar motility. We extracted mucosa from a pig small intestine and characterized its viscoelastic properties to examine the physical challenge motile bacterial pathogens have to overcome to reach the epithelial tissues from the intestinal lumen. Unprocessed intestinal mucus is a viscoelastic hydrogel with a pore size estimated to be between 200 nm and 1 μm from our microrheological analyses and previous imaging (28, 40). V. cholerae is small enough to swim through mucus using flagellar motility. However, many cells were trapped in the mucin matrix and the effective diffusion coefficient of free-swimming cells was severely reduced compared to swimming in liquid.

Previous studies suggested that secreted proteases help V. cholerae colonize the intestinal mucus layer by degrading mucins (63, 64). Under the conditions we tested, incubation of V. cholerae in unprocessed porcine intestinal mucus did not produce measurable changes in the mucus rheological properties, suggesting that secreted proteases are not required during the initial stages of infection when the number of V. cholerae organisms is low. Another study proposed that V. cholerae shears or loses its flagellum in the presence of bovine mucin and initiates the expression of virulence factors (42). In this study, we found that V. cholerae rapidly dies in bovine mucin solutions unless dissolved in rich media (likely quenching an unidentified toxic compound). Dead cells showed the expected Brownian motion, consistent with previous observations (42). We found that V. cholerae can grow in unprocessed porcine intestinal mucus and that the motile behavior stays steady, suggesting that the integrity of the flagellum is not compromised. These results indicate that, besides the physical interactions with the mucus matrix, there were no measurable biological interactions between V. cholerae and mucus under our experimental conditions.

The diffusion coefficient we observed for motile V. cholerae in mucus is sufficient for cells to reach epithelial tissues during infection of the human small intestine. Previous studies have indicated that directional motion controlled by chemotaxis is not required for V. cholerae to infect the host (26, 65, 66). Therefore, V. cholerae is likely performing a diffusive random walk through the mucosa. The typical thickness of mucus in the human small intestine is on the order of a few hundred micrometers and grows about 240 μm per hour (28). The typical first-passage time of a diffusive trajectory can be calculated as the square of the distance to cross divided by twice the diffusion coefficient (67). From our results, we estimate that the typical time V. cholerae would take to penetrate 400 μm of the small intestine mucosa at pH 8 is about 2 h, which is comparable to the time it takes to grow mucosa of that thickness. Therefore, in the absence of factors that interfere with flagellar motility, V. cholerae is intrinsically capable of overcoming the physical barrier formed by intestinal mucus using flagellar motility even without a chemotactic response.

The dynamics of infection of the human small intestine by V. cholerae has not been firmly established, partially because of the limitations of existing animal models (68). The early infection steps may differ significantly between animal models and humans. Studies done on infant rabbits and mice indicate that in the early stage of infection, planktonic V. cholerae cells are distributed throughout the small intestine. The bacterial load then drops in proximal and medial small intestine while the surviving cells preferentially colonize the distal small intestine (26, 69). Only a small fraction of cells can penetrate the mucus layer protecting epithelial tissues. In the later stage of the infection, V. cholerae repopulates all parts of the small intestine (69, 70). Previous studies provided conflicting evidence supporting the role of flagellar motility during infection (20). Some studies found that nonmotile cells are less infectious (71, 72), while others reported that there is no difference and that nonmotile cells can reach the epithelial crypts in infant mice (26). Therefore, the route to the epithelium may vary between experimental models.

The pH gradient along the length of the small intestine may contribute to the preferred site of infection for V. cholerae. In humans, the proximal small intestine is slightly acidic (pH 6.3 to 6.5), while the distal part is slightly alkaline (pH 7.5 to 7.8) (39, 73). V. cholerae can grow between pH 6.5 and 9, but its preferred pH is that of seawater, at ∼8 (74). Acidic pH regulates the expression of virulence factors in V. cholerae. The production of cholera toxin and toxin-coregulated pili is maximal at pH 6.6 (75, 76). Our results showed that MshA affected the motility of El Tor C6706 at acidic pH when grown on soft agar but did not have measurable effect in porcine intestinal mucus, consistent with the previous observation that MshA likely is not involved in host infection (77). On the other hand, high gastrointestinal pH increases the susceptibility of V. cholerae infection (78), and lactic acid-producing bacteria, such as Lactococcus lactis, provide some protection against V. cholerae infections (79).

Our results showed that alkaline pH increases swimming speed and improves the ability of V. cholerae to penetrate intestinal mucus. Because V. cholerae’s flagellar rotation is powered by the transmembrane sodium gradient, the effect of environmental pH on flagellar motility is likely indirect. The main sodium pump of V. cholerae, Na^+^-NQR, has increased activity at alkaline pH and no activity at acidic pH, thereby affecting the sodium potential across the membrane (60, 61). In this study, inhibiting Na^+^-NQR with HQNO had the same effect as reducing pH on motility, presumably because the sodium motive force is weakened. In addition, a previous study reported that a mutant strain lacking NqrA (a subunit of the Na^+^-NQR complex) is defective at colonizing infant mice (80), and inhibiting Na^+^-NQR activity decreased the production of cholera toxin (81). Our model is that V. cholerae has difficulty maintaining a strong sodium motive force at acidic pH, reducing the cells’ capacity to penetrate mucus and reach the epithelium. In addition, acidic pH reduces the production of cholera toxin, which is essential to disrupt the normal function of the small intestine to provide a competitive advantage to V. cholerae. Therefore, the preferred site of infection of V. cholerae in the human small intestine is likely in the ileum, where the pH is alkaline.

## MATERIALS AND METHODS

### Bacterial strains.

V. cholerae strains used in this study were El Tor C6706str2 (82) and Classical O395 (83) biotypes. Our wild-type El Tor strain has a functional *luxO* gene. Strains were fluorescently labeled with the expression of the green fluorescent protein expressed from a constitutive cytochrome *c* V. cholerae promoter on a p15a plasmid derivative (gift from Christopher Waters). The inactivation of *mshA* in the El Tor C6706 background was generated by recombining genomic DNA of mutant EC4926 from the defined transposon mutant library (84) using natural transformation (85). The El Tor *flrA* mutant was generated from previous work (54).

### Growth conditions.

M9 minimal salts (52 mM Na_2_HPO_4_, 18 mM K_2_HPO_4_, 18.69 mM NH_4_Cl, 2 mM MgSO_4_) were supplemented with 10 μM FeSO_4_, 20 μM C_6_H_9_Na_3_O_9_, and 36.4 mM sodium pyruvate. The pH of the growth medium was adjusted with HCl to the desired value. V. cholerae was grown shaking (200 rpm) in liquid cultures at 37°C. Kanamycin was added to 50 μg/ml when needed. For all experiments, V. cholerae cultures were sampled at early stationary phase (1.9 × 10^9^ CFU/ml). Soft-agar plates were prepared with the same medium with the addition of 0.1% (wt/vol) tryptone and 0.3% (wt/vol) Bacto agar (BD). Plates were inoculated with 5 μl of saturated liquid culture (5.8 × 10^6^ cells) on the agar surface and incubated at 37°C for 12 h before measuring colony size.

### Mucus preparation.

Small intestines were obtained from a freshly slaughtered adult pig at the Meat Lab at Michigan State University (USDA permit number 137 from establishment number 10053). The animal was slaughtered as part of the normal work of the abattoir according to the rules set by the Michigan State University Institutional Animal Care and Use Committee (IACUC). The small intestines were acquired from the abattoir with prior consent. The mucosa was gently scraped from the medial part of small intestine and frozen in liquid nitrogen before storage at –80°C. For each experiment, mucus samples were warmed to 37°C and equilibrated for 1 h in a 10-volume excess of M9 salts buffered to the desired pH. Bovine submaxillary gland mucin (M3895; Sigma-Aldrich) solution was prepared at 3% (wt/vol) in LB medium adjusted to pH 8.0 with sodium hydroxide. Nonsoluble particles were separated from the preparation by centrifugation at 21,130 relative centrifugal force (rcf) for 10 min. Porcine stomach (M2378; Sigma-Aldrich) mucin solution was prepared at 3% (wt/vol) in M9 salts at pH 8.0. The survival rate of V. cholerae in bovine submaxillary gland mucin was calculated by enumerating colonies on LB agar plates supplemented with 50 μg/ml kanamycin. Fluorescent beads were added to the samples at 0.15% (wt/vol) and gently mixed.

### Single-cell tracking.

V. cholerae cells were tracked in liquid medium by following the protocol previously described (86). Briefly, V. cholerae cells in the early stationary growth phase were diluted to 1.9 × 10^7^ cells/ml in fresh medium adjusted to pH 6, 7, or 8. Cells were incubated with shaking at 37°C for 15 min before tracking to allow for the adaptation of the chemotaxis response. Polyvinylpyrrolidone (PVP) was added at 0.05% (wt/vol) to the samples to prevent attachment on the glass slide. Six microliters of each sample was dropped on a glass slide and trapped under a 22- by 22-mm number 1.5 coverslip sealed with wax and paraffin to create a thin water film (10 ± 2 μm) for video microscopy. For tracking in mucus or low-melting-temperature agarose, a 130-μm spacer was added between the slide and the coverslip, and fluorescently labeled cells were used. The samples were kept at 37°C during tracking. Images of swimming cells were recorded using an sCMOS camera (Andor Zyla 4.2; Oxford Instruments) at 20 frames per second using a 40× objective (Plan Fluor 40×; Nikon Instruments, Inc.) mounted on an inverted microscope (Eclipse Ti-E; Nikon Instruments, Inc.). Cells were illuminated using phase contrast in liquid or epifluorescence in mucus and agarose. Images were analyzed to detect and localize cells using custom scripts (86), and cell trajectories were reconstructed using the μ-track package (87). The analysis and plots of the cell trajectory statistics were done in MATLAB (The Mathworks, Inc.) as previously described (86).

### Passive microrheology of mucus and agarose gel.

The viscoelasticity of mucus and agarose were measured by tracking the passive diffusion of 1-μm fluorescent polystyrene beads (F8814; ThermoFisher Scientific). To prevent electrostatic or hydrophobic interactions between the beads and the gels, beads were coated with polyethylene glycol (PEG; molecular weight, 2,000 Da). Coating was done by cross-linking carboxyl groups on the surface of the beads with diamine-PEG by following the previously described protocol (88). Beads (0.5%, wt/vol) and Triton X-100 (0.01%, wt/vol; Sigma-Aldrich) were added to samples and mixed gently. Epifluorescence signals from the beads were recorded using an sCMOS camera (Andor Zyla 4.2; Oxford Instruments) at 100 frames per second using a 100× objective (Plan Fluor 100×; Nikon Instruments, Inc.) and a 1.5× multiplier mounted on an inverted microscope (Eclipse Ti-E; Nikon Instruments, Inc.). Images were analyzed to detect and localize beads using custom scripts, and trajectories were reconstructed using the μ-track package (87). The bead trajectories were manually inspected to remove artifacts and erroneous linking. Systematic drift of the trajectories was corrected prior to calculating the bead average mean squared displacement (MSD) and velocity autocorrelation (VAC) as a function of time. The VAC was fitted to a degree six polynomial multiplied by an exponential decay function. The VAC function was integrated according to the Green-Kubo relation (89, 90) to obtain a function that can also be fitted to the MSD with the same parameters. The VAC and MSD were fitted simultaneously using nonlinear least-square regression to separate the dynamic properties of the beads from the tracking noise. The fitted parameters were then used to calculate the storage and loss moduli of the sample according to the generalized Stokes-Einstein equation (91). The analysis and plots of the bead diffusive behavior were done in MATLAB (The Mathworks, Inc.).

### Growth rate analysis.

The growth rates of bacterial cultures were calculated by recording the change in optical density at 590 nm of 200-μl cultures in 96-well plates (CLS3595; Corning) using a Sunrise plate reader (Tecan Trading AG, Switzerland). Cultures were inoculated with 1.6 × 10^6^ CFU/ml in the exponential growth phase and incubated at 37°C with intermittent shaking every 10 min for 24 h. Precautions were taken to limit evaporation.

### c-di-GMP quantification.

The concentration of c-di-GMP was measured as previously described (92). Briefly, 2 × 10^8^ cells sampled during the exponential growth phase were collected on a polytetrafluoroethylene membrane filter (0.2 μm) from each condition. Membranes were submerged and mixed in extraction buffer (40%, vol/vol, acetonitrile, 40%, vol/vol, methanol, 0.1 N formic acid) for 30 min. The extraction solution was spiked with a known amount of N^15^-labeled c-di-GMP to normalize sample loss across samples during extraction. Nonsoluble cell debris was separated by centrifugation (21,130 rcf for 2 min). The soluble fractions were dried in vacuum overnight and resuspended in 100 μl distilled water prior to identification and quantification using mass spectrometry (Quattro Premier XE mass spectrometer; Waters Corp.). c-di-GMP and N^15^-labeled c-di-GMP were detected simultaneously at *m/z* 699.16 and *m/z* 689.16, respectively.

### Statistical analyses.

The statistical significance of the different effects was calculated using Bayesian sampling of linear mixed-effect models, taking into account experimental treatments and random effects from replication. The effect of pH on motility in mucus and in liquid was modeled as response ≈ strain × pH + (1|replicate) using a log-normal link function. The addition of HQNO was modeled as an additional interaction, with concentration modeled as a monotonic relationship. The effect of pH on motility in soft agar and growth rate was modeled as response ≈ strain × pH + (1|replicate) using a normal link function. Models were compiled and sampled using the RSTAN (93) and BRMS packages (94, 95) in R (96). The plots were generated using the ggplot2 (97) and tidybayes (98) packages.

## Supplementary Material

Supplemental file 1

Supplemental file 2
