# Supplementary material for: Alkaline pH Increases Swimming Speed and Facilitates Mucus Penetration for Vibrio cholerae
Source: J Bacteriol. 2021 Mar 8;203(7):e00607-20. doi: 10.1128/JB.00607-20 (PMC8088521; doi:10.1128/JB.00607-20)
Supplement: Supplemental file 1 [file JB.00607-20-s0001.pdf]

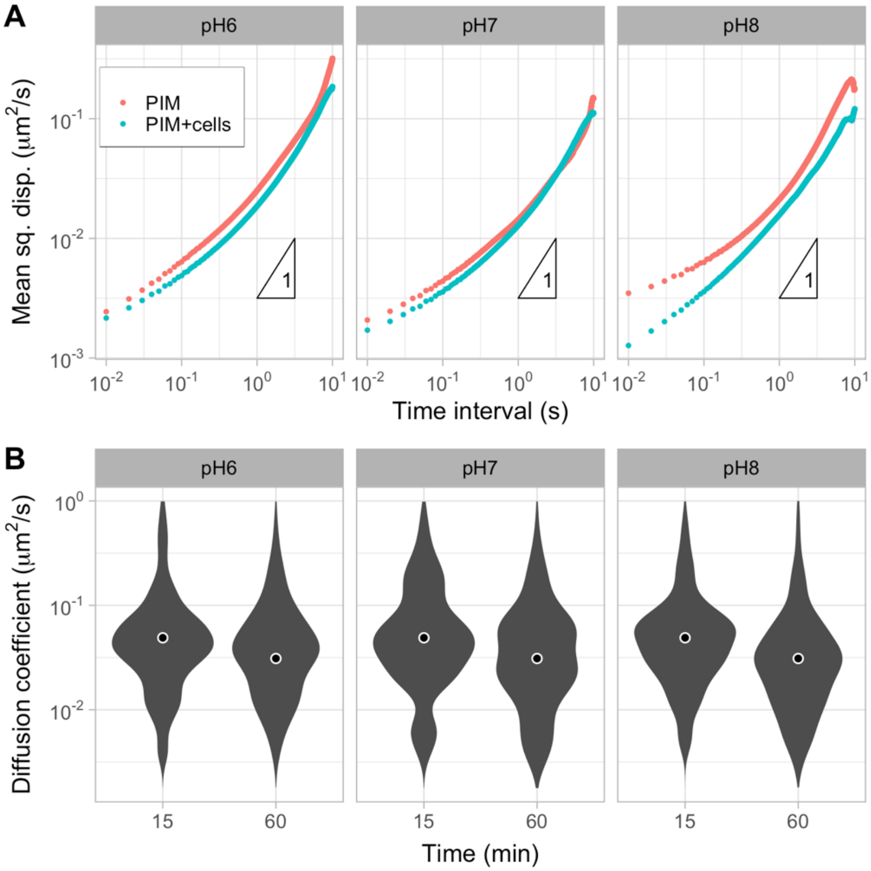

Figure S1. Passive microrheology of porcine intestinal mucus. (A) Mean-squared displacement (Mean sq. disp.) of PEG-coated 1 μm polystyrene beads with respect to time at different pH and after incubation with *V. cholerae* (indicated by different colors). The data points (circles) are the average of trajectories from 2 to 6 replicates (8 to 25 individual trajectories). (B) Distributions of the diffusion coefficient of non-motile *V. cholerae* (*flrA*) after incubation in mucus at different pH. Each distribution represents 3 to 6 replicates combining between 500 and 2,000 individual trajectories. Circles: means.

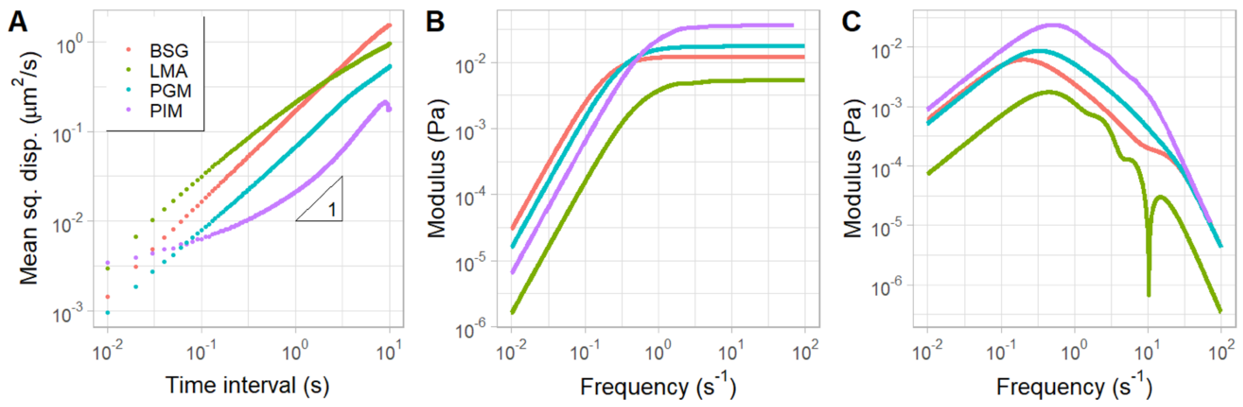

Figure S2. Passive microrheology of mucin solutions. (A) Mean-squared displacement (Mean sq. disp.) of PEG-coated 1 µm polystyrene beads with respect to time in different mucin or agarose preparations (BSG: commercial bovine submaxillary gland mucin, PGM: commercial porcine gastric mucin, PIM: unprocessed porcine intestinal mucus, LMA: low-melting temperature agarose). The data points (circles) are the average of trajectories from 3 to 6 replicates (12 to 25 individual trajectories). A polynomial fit the data (line) was used to calculate the storage and loss moduli using the generalized Strokes-Einstein relation. (B) Storage moduli (elasticity) of different mucin or agarose preparations. (C) Loss moduli (viscosity) of different mucin or agarose preparations.

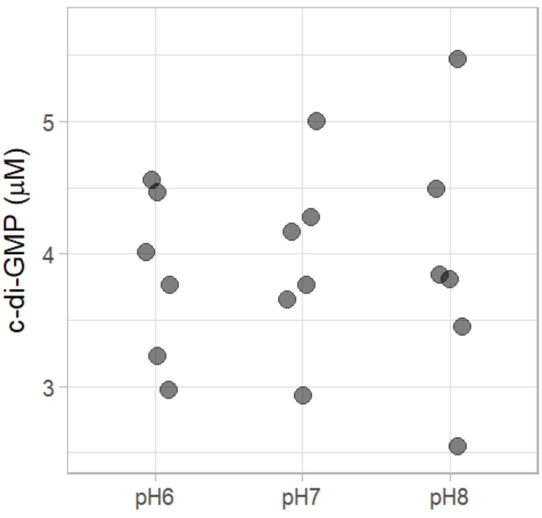

Figure S3. c-di-GMP concentrations in *V. cholerae* El Tor C6706 incubated at different pH.
